# Fractionation of trace elements and human health risk of submicron particulate matter (PM1) collected in the surroundings of coking plants

**DOI:** 10.1007/s10661-017-6117-x

**Published:** 2017-07-11

**Authors:** Elwira Zajusz-Zubek, Tomasz Radko, Anna Mainka

**Affiliations:** 1 Department of Air Protection, Silesian University of Technology, 22B Konarskiego St., 44-100 Gliwice, Poland; 2 Institute for Chemical Processing of Coal, 1 Zamkowa St., 41-803 Zabrze, Poland

**Keywords:** PM1, Trace elements, Bioavailability, Coking plants, Chemical fractionation, Health risk

## Abstract

Samples of PM1 were collected in the surroundings of coking plants located in southern Poland. Chemical fractionation provided information on the contents of trace elements As, Cd, Co, Cr, Hg, Mn, Ni, Pb, Sb and Se in all mobile (F1–F3) and not mobile (F4) fractions of PM1 in the vicinity of large sources of emissions related to energochemical processing of coal during the summer. The determined enrichment factors indicate the influence of anthropogenic sources on the concentration of the examined elements contained in PM1 in the areas subjected to investigation. The analysis of health risk for the assumed scenario of inhabitant exposure to the toxic effect of elements, based on the values of the hazard index, revealed that the absorption of the examined elements contained in the most mobile fractions of particulate matter via inhalation by children and adults can be considered potentially harmless to the health of people inhabiting the surroundings of coking plants during the summer (HI < 1). It has been estimated that due to the inhalation exposure to carcinogenic elements, i.e., As, Cd, Co, Cr, Ni and Pb, contained in the most mobile fractions (F1 + F2) of PM1, approximately four adults and one child out of one million people living in the vicinity of the coking plants may develop cancer.

## Introduction

Epidemiological studies indicate that negative health effects are not only observed in the case of exposure to increased mass concentrations of particulate matter but also result from the chemical, physical and biological properties (Shaughnessy et al. 2015; Zhai et al. 2014). The negative impact is enhanced in the case of exposure to particles with a diameter below 1 μm (PM1), which may cause long-term and cumulative effects in the form of chronic bronchitis, cardiovascular diseases, lung cancer or even death (Laden et al. 2000; Massey et al. 2013; Pope et al. 2015; Samoli et al. 2014). Additionally, particles smaller than 1 μm can directly penetrate into human pulmonary alveoli, where the absorption capacity for the majority of elements contained in the particles reaches 60–80% (Fernández Espinosa et al. 2002; Infante and Acosta 1991), which leads to enhanced negative health effects (Wichmann et al. 2010). Statistical data indicates that on a global scale, approximately 2.1 million deaths annually result from exposure to fine dust (Ahmed et al. 2015; Guha et al. 2015). Previously, conducted investigations into the environment and exposure to particulate matter mainly concern mass concentrations and the total contents of trace elements in different fractions of particulate matter (Kulshrestha et al. 2009; Malaguti 2015; Minguillón et al. 2012; Putaud et al. 2010; Vossler et al. 2015). A large part of these element emissions are produced by the industrial energochemical processing of coal (pyrolysis, gasification) and the processes involving its use for energy purposes (Cao et al. 2014; Dai et al. 2012; Gorka 2002; Kowol et al. 2007; X. Li et al. 2005).

Coal pyrolysis, which is a basic technological process in the coke-making industry, leads to the release of hazardous elements, with volatile products and their distribution among coke, gaseous and liquid products, as well as volatile streams of contaminations that uncontrollably escape from coke oven batteries (Raclavská et al. 2015; Zajusz-Zubek and Konieczyński 2003). The considerably differentiated contents of trace elements, the various chemical and mineral forms in which they occur during the production of coke applied in coking plants is the main factor influencing the level of emissions of dust from the coal pyrolysis process. Emissions of hazardous elements contained in the dust depend also on the age, technology and technical equipment of coke oven batteries. However, coke oven batteries are not solely responsible for emissions from the coal pyrolysis process. The major sources are usually accompanied by fugitive emissions from auxiliary operations, such as the storage, transport and reloading of coal and coke, the management of byproducts and post-production waste. Despite the progressing hermetization of the majority of unit processes, it can be presumed that each of the aforementioned sources plays a part in the increased concentration of particulate matter in coking plant surroundings, which may have a negative impact on inhabitant health.

The results of research obtained in the process of determining the total content of heavy metals in particulate matter and in the respirable fraction are insufficient to define their toxicity and bioavailability. It is only the use of a sequential analysis that enables the estimation of the percentage share of the mobile forms of elements threatening the ecological safety. The availability of scientific data on the forms of occurrence and characteristics of trace elements in dust fractions, especially the fractions including fine and ultrafine particles from the emissions released by coking plants, is very limited.

Fractionation is one of the major methods for evaluating the mobility, bioavailability and potential health risks of a group of compounds of a given element with particular properties, making it possible to forecast the conditions in which secondary contamination of ecosystems that adversely influence ecological safety may occur. Chemical fractionation allows defining the mobility of elements (including heavy metals) in the environment. Sequential extraction schemes differ due to the conditions in which they are carried out and the extracting agents applied (Schleicher et al. 2011; Smichowski et al. 2005; Tessier et al. 1979).

Bioavailability and toxicity define the way in which the chemical form of a trace element influences the environment and human populations. Elements in dust particles are bound in chemical forms characterized by various levels of bioavailability, which, depending on the physical and chemical conditions, can be released and re-incorporated into the natural environment, thus posing a threat to man. Bioavailable forms of hazardous elements, in particular the elements that are soluble in water, should be subject to thorough investigations due to their threat to the environment and human health. According to literature reports, these elements are almost completely bioavailable (Voutsa and Samara 2002; Yadav and Satsangi 2013). Additionally, toxic elements contained in fractions soluble in water probably tend to accumulate on the surface of dust particles; hence, they tend to dissolve (Yadav and Satsangi 2013).

The bioavailable fractions of these elements, especially the elements that are highly soluble in water, easily migrate from the air to the water or soil and penetrate into the human body. Dust particles and elements contained in them easily penetrate into organisms, mainly via airways but also through the alimentary track and skin (Canepari et al. 2014; Hieu and Lee 2010; H. Li et al. 2015; Pui et al. 2014; Wcisło et al. 2002).

Despite the reduction of emissions in recent decades, the presence of carcinogenic and possibly carcinogenic elements in mobile forms enhances the threat to people living near coking plants. This is directly related to the processes of coal energochemical processing. The evaluation of mobile and not mobile forms of elements in relevant fractions of particulate matter enables the description of the potential threats, which result from the migration of particular forms of trace elements contained in dust, mainly via respiratory tracts.

The global coke-making industry has a slightly increasing trend, whereas in Poland it has been maintained at a relatively stable level. Therefore, the significance of investigations undertaken in this work goes beyond the domestic issues. The high concentration of aerosols containing particular chemical substances, including carcinogenic metals, may lead to health problems and an increased death rate for people living in such regions (Dolk et al. 1999; Du Four et al. 2005; García-Pérez et al. 2010). The results of the research are essential for the better identification of threats, especially in the areas near coking plants that are inhabited by several hundred thousand people.

In this work, the researchers have examined the mobile forms of elements (As, Cd, Co, Cr, Hg, Mn, Ni, Pb, Sb and Se) that define their bioavailability and toxicity in PM1 in the surroundings of coking plants. Specifically, this paper includes the following aspects: (1) a comparative analysis of the PM1 levels in the surroundings of four individual working coking plants; (2) the enrichment factors (EF); (3) a chemical fractionation of the trace elements in PM1; and (4) parameters necessary to quantitatively evaluate inhalation exposures, such as risk assessment code (RAC), hazard quotient (HQ), hazard index (HI) and excess lifetime cancer risk (ELCR) factors, will be developed in the exposure assessment.

## Material and methods

**Fig. 1:**
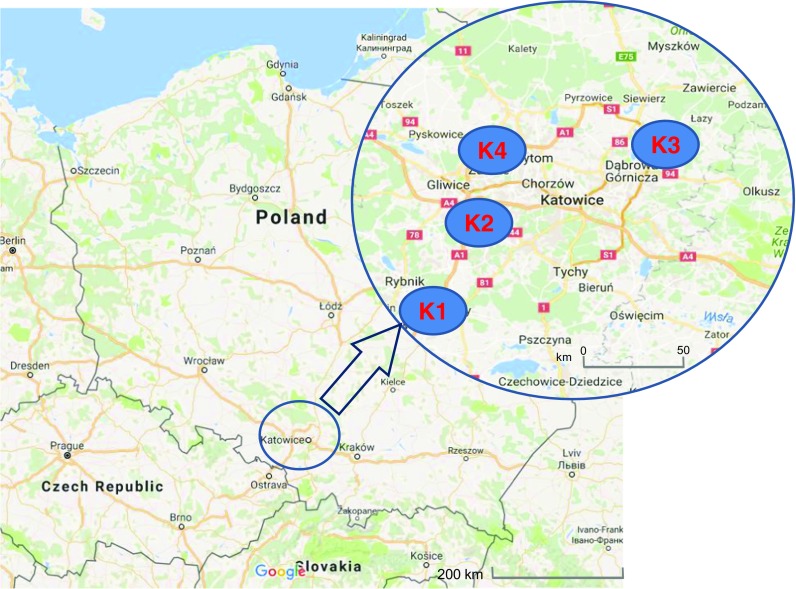
Localization of sampling sites (map data: ©2016 GeoBasis-DE/BKF (©2009), Google)

Measurement points were located in southern Poland, in the vicinity of four selected coking plants. The collection points near the coking plants (K1, K2, K3 and K4) were situated within a distance of ca 2 km to the north-east of the relevant facility (Fig. 1). The location of measurement points was a compromise, taking into consideration the representativeness of the recipient as well as the possibilities of connecting the testing equipment and the permission of the estate owner. Due to the need of eliminating the influence of the heating season, especially the low emission, presented in the research conducted by Zajusz-Zubek et al. (2015a), the measurement sessions were carried out only in the summer period. The methodology adopted here follows that of Zajusz-Zubek et al. (2015b).

### Location of measurement points

Samples of particulate matter were taken in weekly periods, with four sessions at one point. Measurement sessions in the vicinity of four working coking plants were carried out from 4 May to 28 August 2015. The first point, K1, was in Czerwionka-Leszczyny (50°10′11.36″ N; 18°40′34.35″ E) in the community of Leszczyny, Silesian Voivodship, in the vicinity of a small working coking plant (1 battery working in the stamp charging system with a coke wet-quenching facility). The town is inhabited by 27,300 people. The second point, K2, was situated in Popielów (50°3′19.76″ N; 18°30′21.69″ E), a suburban district of Rybnik in Silesian Voivodship. It is situated in the vicinity of a small working coking plant (1 battery working in the stamp charging system with wet-quenching of coke, newly built in 2008), with 33,000 inhabitants. The third point, K3 (50°21′24.08″ N; 19°21′37.46″ E), was situated in a rural area, in Łęka—a district of Dąbrowa Górnicza, Silesian Voivodship, in the vicinity of a large coking plant (5 batteries working in the top charging system with a coke dry-quenching facility that serves 4 coke oven batteries and a coke wet-quenching facility serving 1 coke oven battery). The district has 700 residents. The fourth point, K4 (50°21′0.47″ N; 18°53′15.44″ E), was situated in Bytom, a town with 174,700 inhabitants in Silesian Voivodship. On the outskirts of the town, there is a small coking plant (1 battery working in the stamp charging system with a coke wet-quenching facility). All the points in the vicinity of the selected working coking plants were located in the territory of the industrialized macro-region of Upper Silesia (Fig. 1).

The pollution of air with particulate matter in the area investigated in this study is influenced by various local sources of emissions. In measurement points K1, K2, K3 and K4, emissions primarily accompany the coal coking processes, as well as emissions released in auxiliary processes, i.e., the deposition or transport of coal, production and post-production waste management. Moreover, the level of air pollution is also determined by emissions from industrial plants in this area and low sources as well as from the processes of solid fuel combustion for communal purposes and vehicular emissions.

### Sample collection

Samples of submicron particulate matter (PM1) were collected using a Dekati® PM10 cascade impactor, produced by Dekati (Finland), with an air flow intensity of 1.8 m^3^/h. The Dekati® PM10 impactor guarantees the collection of particulate matter samples for three cut-off diameters: 10, 2.5 and 1 μm. PM1 was collected on a Teflon filter, on which particles having a diameter ≤ 1 μm (Pall Teflo R2PJ047, diameter of 47 mm, Pall International Ltd., New York, NY, USA) are deposited. The average volume of air flowing through the filters reached ca. 300 m^3^. The impactor’s capture efficiency was characterized by an uncertainty below 2.8%. The mass of dust collected at particular impactor stages was determined by the gravimetric method and referred to the volume of air passed (μg/m^3^).

Samples were collected at a height of 1.5 m from the ground surface, that is, in a breathing zone. PM1 was collected on 7-day cycles (16 weeks) in the vicinity of four working coking plants. The measurement campaign encompassed four measurement sessions, which were separate for each measurement point. One session included the collection of PM1 samples and blank filters.

### Chemical fractionation

The PM1 collected on the relevant stage of the Dekati® PM10 cascade impactor was subjected to chemical fractionation. Subsequent stages of the investigations involved the chemical fractionation of the collected PM1. The research was preceded by literature studies regarding the fractionation methodology, which showed differences between the conditions of the process and the extractants applied for various matrixes. One of the speciation analysis schemes for particulate matter is the analysis proposed by Tessier et al. (1979), involving division into relevant fractions. This scheme was modified by Fernández Espinosa et al. (2002), Sanchez-Rodas et al. (2012) and Schleicher et al. (2011), as well as in a publication presenting the results of a speciation analysis of trace elements contained in PM1 collected in the surroundings of coal-fired power stations.

The adopted scheme takes into consideration the presence of elements in the following chemical fractions: exchangeable—the most bioavailable (F1)—*highly mobile fraction*; related to carbonates and oxides (F2)—*mobile fraction*; related to organic matter (F3)—*less mobile fraction*; and permanently bound with minerals (F4)—*not mobile fraction*. An analysis according to the adopted scheme involves the chemical fractionation of particulate matter in the process of sequential extraction with solutions of an increasing leaching power (Zajusz-Zubek 2016; Zajusz-Zubek et al., 2015a). Such a procedure enables the simulation of the natural and anthropogenic environmental conditions.

The solutions that were obtained from the four extraction steps (F1–F4) were filtered using the DigiFILTER system (PerkinElmer, Inc., Waltham, MA, USA; 0.45 μm). Subsequently, for every fraction, the As, Cd, Co, Cr, Hg, Mn, Ni, Pb, Sb and Se content was determined. The total content of each trace element was calculated as the sum of the four extraction steps (Fernández Espinosa et al. 2002; Richter et al. 2007).

### Chemical analysis

The qualitative-quantitative analysis of the solutions obtained for particular fractions was conducted by the method of inductively coupled plasma mass spectrometry, using ICP-MS (NexION 300D, PerkinElmer, Inc., Waltham, MA, USA) equipment. For all the simultaneously determined elements, the parameters of the equipment work were the same as those quoted in publication (Zajusz-Zubek et al., 2015b).

Certified multi-element standard solutions of 1000 μg/cm^3^ (CertPUR ICP multi-element standard solution VI for ICP-MS, produced by Merck, Germany) were used as calibration solutions for determining ^75^As, ^111^Cd, ^59^Co, ^53^Cr, ^200^Hg, ^55^Mn, ^60^Ni, ^206^Pb, ^121^Sb and ^82^Se. All the samples were measured in tenfold repetitions. The determined quantification limits were based on 10 independent measurements for a blind experiment. The average value and the value of standard deviation *SD* were calculated for the results obtained in this way.

The correct determination of element contents was verified with certified reference materials: the European Reference Material ERM®-CZ120 and the Standard Reference Material SRM 1648a (National Institute of Standards and Technology, USA). The recovery with the aforementioned certified materials reached 111% for ERM®-CZ120 and 96% for SRM 1648, Cd (97 and 105%), Co (108 and 97%), Cr (103 and 94%), Mn (106 and 100%), Ni (107 and 102%), Pb (107 and 105%) and Sb (99 and 91%), respectively. The certified materials did not contain Hg and Se.

### Statistical analyses

All of the statistical analyses regarding the determined concentrations of PM1 and the elements were conducted using the statistical package Statistica 10 (StatSoft, Tulsa, OK, USA). The adopted level of significance for observing the statistically significant differences was *p* < 0.05.

## Results and discussion

### Concentrations of PM1

**Fig. 2:**
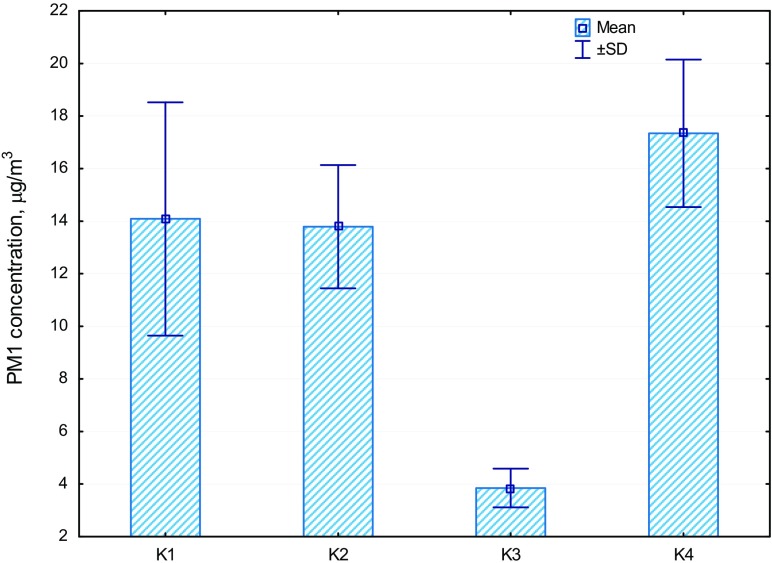
Concentration of PM1 in the surroundings of the selected coking plants (K1–K4)

The variability of PM1 concentrations in the surroundings of the four selected working coking plants has been presented in Fig. 2. The average PM1 concentration obtained as a result of the investigations conducted in the vicinity of the coking plants (Fig. 2) reached 12.17 μg/m^3^ (ranging from 3.08 to 26.37 μg/m^3^). Among all the examined locations, the lowest average PM1 concentration in μg/m^3^ was observed at site K3—3.85 ± 1.27, whereas the highest concentration was noted at location K4—17.35 ± 4.86.

### Total content of elements in PM1

Within the framework of the conducted investigations, the researchers also determined the total concentration of elements (As, Cd, Co, Cr, Hg, Mn, Ni, Pb, Sb and Se) in submicron particles, PM1, collected in the surroundings of the coking plants, calculated as a sum of their concentrations in F1–F4 fractions obtained in sequential extraction (H. Li et al. 2015; Palma et al. 2015; Zajusz-Zubek et al., 2015b). Table 1 presents the levels of these elements’ total contents for each of the coking plants separately.

**Table 1:** The total mass concentration of elements in the surroundings of selected coking plants (K1–K4)

Element	Average concentrations (ng/m^3^)			
	K1	K2	K3	K4
As	7.66E−01 ± 1.57E−01	7.56E−01 ± 2.23E−01	1.03E+00 ± 7.11E−02	1.34E+00 ± 4.93E−01
Cd	6.38E−01 ± 5.98E−02	5.85E−01 ± 2.06E−01	1.00E+00 ± 2.87E−01	1.89E+00 ± 2.98E−01
Co	4.71E−02 ± 2.22E−03	1.54E−01 ± 1.58E−01	1.31E−01 ± 8.70E−04	1.78E−01 ± 3.09E−02
Cr	9.74E+00 ± 7.38E−01	9.74E+00 ± 7.58E−02	2.69E+01 ± 1.16E−01	2.74E+01 ± 4.82E−01
Hg	2.47E−01 ± 7.53E−03	2.54E−01 ± 2.54E−03	5.65E−01 ± 1.61E−03	5.69E−01 ± 6.83E−03
Mn	4.90E+00 ± 5.09E−01	1.27E+01 ± 5.73E+00	1.01E+01 ± 2.30E+00	1.18E+01 ± 3.62E+00
Ni	1.63E+00 ± 9.14E−01	9.33E−01 ± 2.54E−01	5.10E+00 ± 4.26E−01	5.50E+00 ± 8.66E−01
Pb	1.62E+01 ± 2.60E+00	1.42E+01 ± 3.95E+00	2.91E+01 ± 4.51E+00	7.40E+01 ± 2.77E+01
Sb	1.49E+00 ± 4.99E−01	9.51E−01 ± 9.80E−02	9.19E−01 ± 2.05E−01	1.84E+00 ± 7.87E−01
Se	3.42E+00 ± 2.54E−01	3.58E+00 ± 1.95E−01	1.03E+00 ± 8.78E−02	1.59E+00 ± 5.30E−01

Among the elements found in the vicinity of the coking plants (Table 1), the highest average total contents of PM1 were noted for Pb and Cr, at 33.34 ± 10.98 and 18.43 ± 9.02 ng/m^3^, respectively. The concentrations of Mn, Ni, Se, Sb and Cd in PM1 reached several ng/m^3^ in PM1, and in the case of As, Hg and Co, they were below 1 ng/m^3^.

The average total content of elements in PM1 in the surroundings of four coking plants decreased in the following order: Pb > Cr > Mn > Ni > Se > Sb > Cd > As > Hg > Co. A comparison of the results of research presented in this work with similar investigations conducted in the surroundings of a large coking plant in China showed considerable differences in the concentrations of the examined elements in PM2.5, reaching even a few orders of magnitude (Cao et al. 2014). Despite a reduction in contamination in recent decades, the presence of Cr, which is a carcinogenic element, and Pb, a possibly carcinogenic element, in fine PM increases the threat posed to people living in the surroundings of coking plants. In this case, information on the forms in which these elements occur—mobile or not mobile—is also of great importance.

### Enrichment factor (EF) analysis

The influence of anthropogenic sources on the concentrations of all trace elements contained in fine PM1 has been calculated on the basis of the so-called enrichment factor, *EF*. The average enrichment factor was calculated from the following equation:

1 $$ {EF}_x=\frac{{\left({c}_x/{c}_{\mathrm{ref}}\right)}_{\mathrm{PM}1}}{{\left({c}_x/{c}_{\mathrm{ref}}\right)}_{\mathrm{lithosphere}}} $$

where *c*_*x*_ and *c*_ref_ are the total contents of element *x* and the reference element in PM1 and in the outer layer of earth’s crust (lithosphere). Crustal elements are usually used as reference elements, among others, Fe (Enamorado-Báez et al. 2015), Al (Cesari et al. 2012; Clements et al. 2014; Hieu and Lee 2010; H. Li et al. 2013; Rita Perrone et al. 2006), Mn (Fabretti et al. 2009; Sakata and Asakura 2011; Zajusz-Zubek et al., 2015a) and Zr (Fabretti et al. 2009). In the presented work, Mn, which typically occurs in the lithosphere, has been selected as a reference element; hence, *EF*_Mn_ = 1. However, the chemical composition of the geological environment in Upper Silesia is not comparable to that of the upper layer of the earth’s crust as estimated by Wedepohl (1995). It is assumed that a value of *EF* close to one clearly indicates the geogenic origin of a particular element. An *EF* value below 10 shows the influence of both natural sources and possible distant anthropogenic sources of emissions on the concentration of this element in the air, whereas a value above 10 reveals the anthropogenic origin of a particular element.

**Fig. 3:**
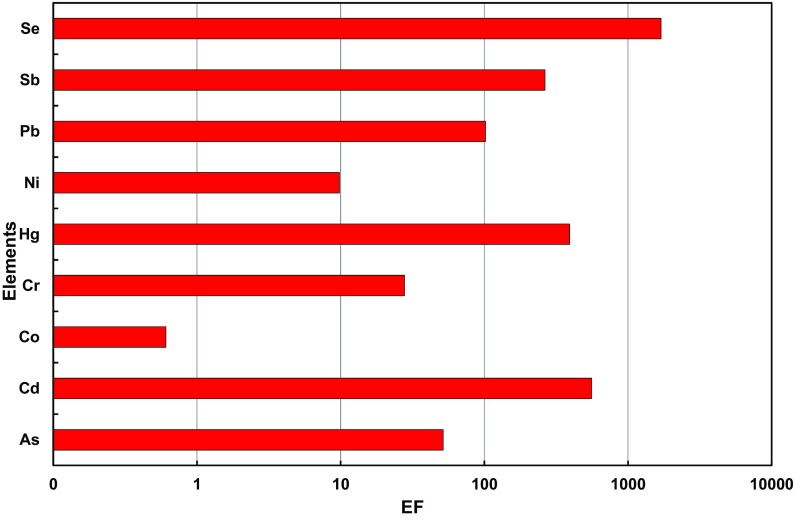
Average enrichment factors (*EF*s) of elements in PM1 in the surroundings of the selected coking plants

The results of investigations interpreted through the average values of enrichment factors, compared to Mn in the surroundings of the working coking plants (K1, K2, K3 and K4), have been presented in Fig. 3. The values of *EF* in the surroundings of four coking plants for PM1 decreased in the following order: Se > Cd > Hg > Sb > Pb > As > Cr > Ni > Co. Elements contained in PM1 were classified into two groups on the basis of their enrichment factor values. Co (*EF* < 1), of crustal origin, was included in group 1, as well as Ni (*EF* = 9.8). The nickel contained in the fraction of PM1 in the surroundings of the coking plants may originate from the re-suspension of crustal and road dust as well as from mixed anthropogenic and geogenic sources. This was also confirmed by the research on PM1 in the surroundings of power stations, presented by the publication author and her co-workers (Zajusz-Zubek et al., 2015b).

Group 2 included As and Cr—moderately enriched elements with enrichment factors ranging from 11 to 100. Also, such elements as Se, Cd, Hg, Sb and Pb, characterized by a high enrichment factor (*EF* > 100) in PM1 in the vicinity of the coking plants, were assigned to this group.

The high values of the enrichment factors indicate the predominance of anthropogenic sources, which influence the concentrations of elements in the air.

### Distribution of trace elements as a result of chemical fractionation

The method of chemical fractionation, based on sequential extraction, enables the determination, in terms of environmental bioavailability, of the mobility of a group of compounds of a particular element contained in PM1. It enables a prediction of conditions that favor the occurrence of ecosystem secondary contamination, which has an adverse impact on ecological safety and human health. Elements contained in fractions (F1–F4) are characterized by various bioavailabilities and biological-chemical activities. In the presented investigations, the concentrations of As, Cd, Co, Cr, Hg, Mn, Ni, Pb, Sb and Se were determined for four fractions F1–F4 of PM1 obtained through sequential extraction (Table 2).

**Table 2:** Concentrations of elements related to selected chemical fractions contained in PM1 in the surroundings of the selected coking plants (K1–K4)

Fraction (ng/m^3^)	As	Cd	Co	Cr	Hg	Mn	Ni	Pb	Sb	Se
	K1									
F1	7.45E−01 ± 1.55E−01	3.79E−01 ± 6.87E−02	2.10E−02 ± 2.34E−03	1.55E−01 ± 3.16E−02	6.71E−02 ± 2.05E−03	2.52E+00 ± 4.14E−01	7.76E−01 ± 9.55E−01	7.50E+00 ± 2.25E+00	7.97E−01 ± 3.38E−01	5.93E−01 ± 1.70E−01
F2	3.92E−03 ± 1.19E−04	8.57E−02 ± 6.56E−03	4.78E−03 ± 5.26E−04	2.47E−01 ± 7.51E−03	2.45E−03 ± 7.46E−05	7.64E−01 ± 5.02E−02	1.37E−01 ± 1.15E−01	5.64E+00 ± 9.70E−01	1.20E−01 ± 4.57E−02	8.20E−02 ± 2.50E−03
F3	1.18E−02 ± 5.64E−03	5.11E−02 ± 3.94E−02	1.14E−02 ± 3.48E−04	1.96E+00 ± 5.64E−01	1.05E−01 ± 3.21E−03	1.15E+00 ± 8.68E−02	1.72E−01 ± 5.24E−03	1.61E+00 ± 4.11E−01	2.66E−01 ± 1.71E−01	2.19E−01 ± 6.68E−03
F4	5.88E−03 ± 1.79E−04	1.23E−01 ± 3.75E−03	9.85E−03 ± 3.00E−04	7.38E+00 ± 2.25E−01	7.23E−02 ± 2.20E−03	4.67E−01 ± 1.15E−01	5.48E−01 ± 5.19E−01	1.41E+00 ± 1.33E+00	3.11E−01 ± 3.24E−01	2.52E+00 ± 7.68E−02
	K2									
F1	7.40E−01 ± 2.23E−01	3.31E−01 ± 1.25E−01	3.40E−02 ± 7.51E−03	2.86E−01 ± 6.88E−02	6.89E−02 ± 6.90E−04	5.49E+00 ± 2.55E+00	3.36E−01 ± 1.27E−01	6.52E+00 ± 6.56E−01	5.56E−01 ± 2.68E−02	6.88E−01 ± 1.69E−01
F2	4.02E−03 ± 4.03E−05	6.03E−02 ± 8.62E−03	7.04E−03 ± 1.08E−03	2.53E−01 ± 2.53E−03	2.51E−03 ± 2.52E−05	1.57E+00 ± 6.00E−01	8.16E−02 ± 1.60E−02	5.78E+00 ± 2.55E+00	8.22E−02 ± 2.24E−03	8.41E−02 ± 8.42E−04
F3	6.02E−03 ± 6.03E−05	8.70E−03 ± 8.71E−05	1.17E−02 ± 1.17E−04	1.63E+00 ± 1.64E−02	1.08E−01 ± 1.08E−03	4.51E+00 ± 2.31E+00	2.65E−01 ± 1.51E−01	9.18E−01 ± 1.89E−01	7.48E−02 ± 1.23E−01	2.25E−01 ± 2.25E−03
F4	6.03E−03 ± 6.04E−05	1.85E−01 ± 1.01E−01	1.02E−01 ± 1.58E−01	7.57E+00 ± 7.58E−02	7.41E−02 ± 7.43E−04	1.11E+00 ± 7.27E−01	2.50E−01 ± 2.51E−03	9.59E−01 ± 6.35E−01	2.37E−01 ± 1.98E−01	2.59E+00 ± 2.59E−02
	K3									
F1	1.01E+00 ± 7.10E−02	7.40E−01 ± 2.59E−01	2.27E−02 ± 1.71E−03	2.48E−01 ± 4.40E−02	3.03E−03 ± 8.64E−06	5.06E+00 ± 1.13E+00	4.92E−01 ± 1.05E−01	1.31E+01 ± 2.40E+00	6.53E−01 ± 1.42E−01	7.20E−01 ± 7.74E−02
F2	3.98E−03 ± 1.13E−05	1.06E−01 ± 2.90E−02	4.31E−03 ± 6.71E−04	2.72E−01 ± 7.74E−04	2.48E−03 ± 7.08E−06	1.03E+00 ± 3.09E−01	7.50E−02 ± 4.67E−02	1.02E+01 ± 1.75E+00	5.28E−02 ± 1.52E−02	5.10E−02 ± 9.77E−03
F3	5.96E−03 ± 1.70E−05	6.79E−02 ± 7.40E−04	2.06E−02 ± 5.88E−05	1.86E+01 ± 5.29E−02	1.13E−02 ± 3.23E−05	2.89E+00 ± 9.13E−01	5.97E−01 ± 3.26E−01	4.38E+00 ± 9.81E−01	1.30E−01 ± 3.44E−02	1.94E−01 ± 5.54E−04
F4	5.96E−03 ± 1.70E−05	9.02E−02 ± 2.57E−04	8.36E−02 ± 2.38E−04	7.81E+00 ± 2.23E−02	5.48E−01 ± 1.56E−03	1.10E+00 ± 3.48E−01	3.93E+00 ± 1.12E−02	1.41E+00 ± 8.64E−02	8.35E−02 ± 2.11E−02	6.40E−02 ± 1.82E−04
	K4									
F1	1.33E+00 ± 4.93E−01	1.43E+00 ± 2.30E−01	4.02E−02 ± 8.55E−03	4.95E−01 ± 1.98E−01	3.06E−03 ± 3.66E−05	5.70E+00 ± 1.62E+00	2.73E−01 ± 3.90E−02	3.37E+01 ± 1.01E+01	1.26E+00 ± 5.10E−01	1.26E+00 ± 5.10E−01
F2	4.01E−03 ± 4.80E−05	2.46E−01 ± 5.61E−02	1.01E−02 ± 2.64E−03	2.74E−01 ± 3.28E−03	2.50E−03 ± 3.00E−05	1.62E+00 ± 7.00E−01	7.02E−02 ± 1.28E−02	2.79E+01 ± 1.54E+01	1.62E−01 ± 8.60E−02	6.52E−02 ± 1.68E−02
F3	6.01E−03 ± 9.33E−02	1.25E−01 ± 4.78E−02	4.29E−02 ± 2.10E−02	1.87E+01 ± 2.24E−01	1.14E−02 ± 1.37E−04	3.54E+00 ± 1.34E+00	1.19E+00 ± 7.77E−01	1.10E+01 ± 5.59E+00	2.12E−01 ± 1.61E−01	1.96E−01 ± 2.35E−03
F4	6.01E−03 ± 7.20E−05	9.09E−02 ± 1.09E−03	8.42E−02 ± 1.01E−03	7.87E+00 ± 9.43E−02	5.52E−01 ± 6.62E−03	9.11E−01 ± 1.09E−02	3.96E+00 ± 4.75E−02	1.37E+00 ± 1.65E−02	2.04E−01 ± 1.01E−01	6.45E−02 ± 7.73E−04

**Fig. 4:**
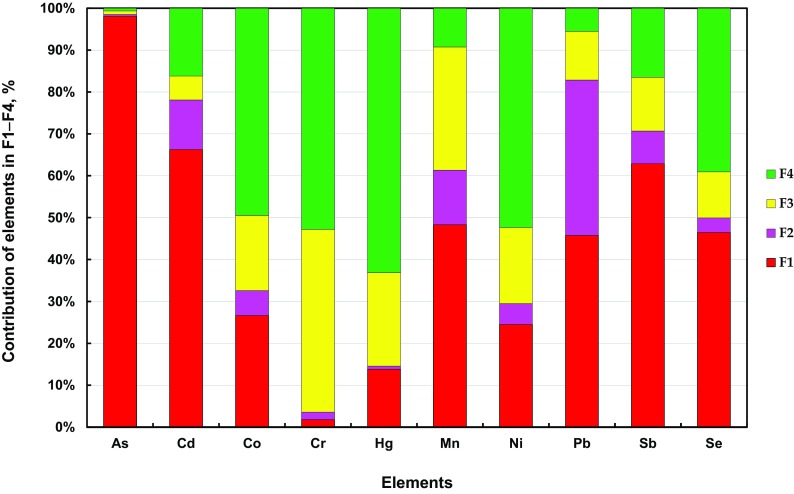
Average contribution of trace elements in the extracted fractions of PM1 collected in the surroundings of the selected coking plants (K1–K4)

Samples for tests were taken in the surroundings of working coking plants. The average percentage contents of the examined elements in fractions F1–F4 extracted from PM1 for all the measurement points (K1–K4) in the surroundings of coking plants have been presented in Fig. 4.

The obtained results revealed differentiation in both the total content of elements in PM1 and their affiliation to particular fractions (F1–F4).

Among the analyzed elements contained in PM1 in the surroundings of coking plants, the highest average percentage contents in the water-soluble fraction, which is most dangerous for the environment (F1), were noted for As (98.08%), Cd (66.27%) and Sb (62.89%) as well as for Mn (48.33%), Se (46.50%) and Pb (45.78%). Moreover, a relatively high content of Pb in the carbonate and oxide fraction (F2) was found in the vicinity of a coking plant, reaching 37.08%. The contents of the remaining elements ranged from a few to several percent in the surroundings of the coking plants (Fig. 4).

It is also noteworthy that fractions F1 and F2 are dominated by elements that the International Agency for Cancer Research (IARC) classifies as carcinogenic—As and Cd, and potentially carcinogenic to humans—Pb, as well as Sb, which probably has carcinogenic properties (Fu et al. 2011).

Due to the lack of literature data on the forms of elements contained in different fractions of particulate matter in the surroundings of working coking plants, a comparison of the elements’ mobility using literature data becomes problematic.

During the investigations into fine (PM2.5) and suspended particulate matter (PM10) in the surroundings of coking plants, the author also confirmed a high content of carcinogenic elements, an average of more than 90 As, 50 Sb and 40% Cd in the water-soluble fraction (F1), which is the most dangerous for the environment. Moreover, in the vicinity of coking plants, a relatively high content of Pb in the carbonate and oxide fraction (F2) in PM2.5 and PM10 was found, reaching 46.36 and 50.49%, respectively. The results of these investigations have been presented in the author’s publication (Zajusz-Zubek 2016). The presence of Pb, mainly in the F2 fraction in total suspended particulates (TSP), was also signaled in the investigations conducted in urban areas in China (Schleicher et al. 2011).

### Impact of particular fractions of carcinogenic and toxic elements on health

The level of concentrations of soluble (mobile) fractions of elements released from aerosol particles is the main indicator of their toxicity. Dust particles easily penetrate into the human organism, mainly via the respiratory tract. After these particles have been inhaled, their surface reacts with physiological fluids, posing a health risk due to the elements contained in the dust (Fu et al. 2011; Guha et al. 2015).

**Fig. 5:**
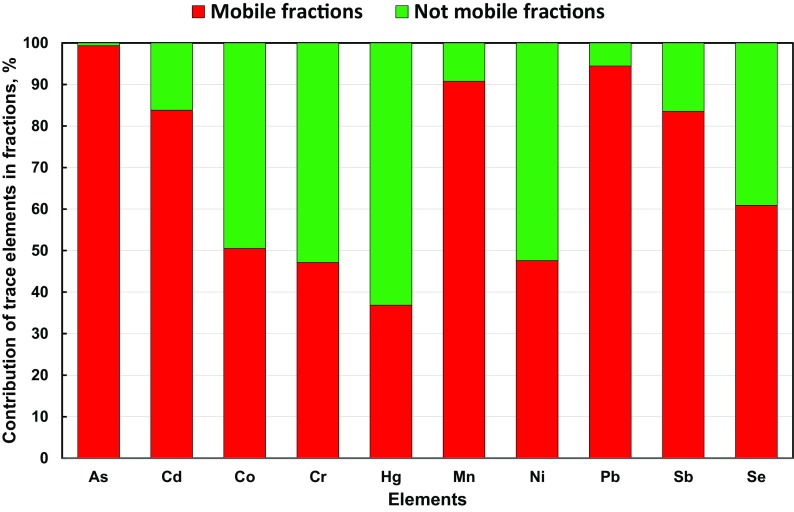
Average contribution of trace elements in the mobile and not mobile fractions in PM1 collected in the surroundings of the selected coking plants (K1–K4)

The most bioavailable elements are contained in the exchangeable fraction (F1), whereas the ones related to carbonates and oxides present in the fraction are referred to as carbonate (F2), and the ones related to organic matter (F3) are treated as mobile forms. The first two fractions, i.e., F1 and F2, cause direct environmental effects, including health risks. Fraction F3 may also cause potential environmental effects and health threats in the event there are conditions enabling its activation; it may also become mobile and migrate easily to fraction F2 or F1. The evaluation of mobile fractions (F1–F3) and the not mobile fraction (F4) with regard to the presence of elements in the examined PM1 samples enables the estimation of potential hazards to human health resulting from the migration of particulate forms of trace elements penetrating into people, mainly via airways. The percentage contents of elements in mobile fractions (F1 + F2 + F3) in the surroundings of selected coking plants for PM1 have been presented in Fig. 5.

The analysis of the contents of the determined elements as their sum in specified mobile fractions of PM1 taken in the surroundings of selected coking plants enabled them to be put into a decreasing order as follows: As (99.35%) > Pb (94.46%) > Mn (90.76%) > Cd (83.83%) > Sb (83.52%) > Se (60.92%) > Co (50.53%) > Ni (47.60%) > Cr (47.18%) > Hg (36.88%). In general, among the examined elements in PM1 in the surroundings of coking plants, the highest percentage content in mobile fractions was noted for As, Pb, Mn, Cd, Sb, Se and Co. Contents below 50% were found for Ni, Cr and Hg.

Elements that have high percentage contents in mobile fractions come from anthropogenic sources, which is indicated by their high enrichment factors. This is also confirmed by literature data presenting the results of other investigations (Slezakova et al. 2014). A high content of As (91.91%), Pb (86.57%) and Cd (77.66%) in PM2.5 mobile fractions in an industrial area in China is also presented by the research of Li et al. (2015). Investigations conducted by Wiseman and Zereini (2014) revealed the high solubility of As and Sb in an artificially prepared body fluid that naturally occurs in human lungs. The author’s research (Zajusz-Zubek 2016) also revealed the highest percentage contents of these elements in the most mobile fraction F1. This may indicate a high health risk due to the content of the listed elements at the selected measurement points.

### Risk of impact of elements on health based on the environmental risk assessment code (RAC)

**Fig. 6:**
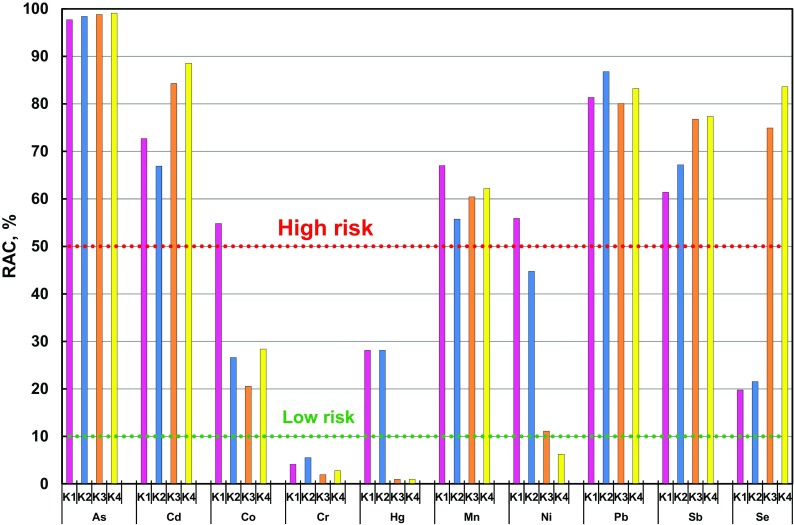
Risk assessment code (RAC) of elements contained in PM1 collected in the surroundings of four coking plants

As mentioned before, the elements considered bioavailable are those that occur in the exchangeable fraction, i.e., the most bioavailable fraction (F1) and the so-called carbonate fraction (F2). For this reason, to evaluate the impact of carcinogenic and toxic elements on the health of inhabitants living in the vicinity of coking plants, the author used the risk assessment code (RAC), taking into account two mobile fractions, i.e., F1 and F2. The author used the data for risk evaluation in accordance with the adopted method of chemical fractionation. The results of the risk assessment based on the RAC for the analyzed elements contained in mobile fractions (F1 + F2) of PM1 taken at measurement points in the surroundings of coking plants have been presented in Fig. 6. The RAC method allows an estimation of the level of threat to the environment from a particular element. No hazard from this element can be expected if only its total content in both fractions, i.e., the exchangeable and so-called carbonate fractions, is below 1%. In the event the content of a particular element in the F1 and F2 fractions ranges from 1 to 10%, the risk is classified as low, from 11 to 30%, as medium and from 31 to 50%, as high. If the content of an element in F1 exceeds 50%, the estimated risk is very high (H. Li et al. 2013, 2015).

In summary, a very high risk at all the measurement points (K1–K4) located in the surroundings of coking plants was noted for the following elements found in PM1: As, Cd, Mn, Pb and Sb, as well as Se (except measurement points K1 and K2), and Co, as well as Ni at point K1. A low threat in all the analyzed points in the vicinity of coking plants was posed by Cr (without taking into consideration a particular degree of oxidation), Hg only at points K3 and K4 and Ni only at point K4. It is worth emphasizing that based on the risk assessment code (RAC), a very high risk has been revealed due to the presence of carcinogenic (As and Cd) and potentially carcinogenic elements (Pb and Sb) in PM1 subjected to analysis at the points located in the surrounding coking plants (Fig. 6).

### Assessment of health risk for people residing in the surroundings of coking plants

Due to the harmful effect of trace elements, including carcinogenic and toxic metals in various mobile forms related to the fractions of particulate matter in atmospheric air, the assessment of health risk is becoming increasingly important. Risk characteristics can be evaluated both qualitatively—by quoting the risk category, i.e., low, medium or high, and quantitatively—by quoting its value, e.g., in the form of hazard quotient (*HQ*) or cancer risk (*ELCR*). The problem with the assessment of health risk due to the impact of mobile forms of toxic and carcinogenic elements in various fractions of particulate matter in the world is raised only sporadically (Fernández Espinosa et al. 2002; Infante and Acosta 1991). In this work, the health risk assessment has been based on a US EPA model (H. Li et al. 2015; Slezakova et al. 2014; Sun et al. 2014; Wiseman and Zereini 2014), which contains an exposure evaluation and risk characteristics. The assessment of the impact of the forecasted exposure to mobile forms of the examined carcinogenic and toxic elements contained in PM1 in the surroundings of coking plants (K1–K4) was conducted on the basis of the results of chemical fractionation based on sequential extraction. The authors assumed that exposure was from the inhalation route of absorbing mobile fractions of carcinogenic and toxic elements with PM1 dust, which is quoted as one of the major routes of exposure to elements contained in PM (Voutsa and Samara 2002; Yadav and Satsangi 2013; Zajusz-Zubek et al., 2015a) and, at the same time, the main route of exposure to dust causing health problems (Delgado et al. 2011; H. Li et al. 2013; Palma et al. 2015; Singh et al. 2005; Sundaray et al. 2011; Teng et al. 2015). People inhabiting the surroundings of the coking plants were divided into two categories: adults and children. Moreover, in accordance with the US EPA, it was assumed that a person might be exposed to mobile fractions of elements contained in fine dust (PM1) in the air for 350 days a year, 24 h a day, during a lifetime of 24 years—for both an adult and a 6-year-old child (US EPA 1989). The averaging period in the case of the scenario of lifelong chronic exposure was 70 years. The carcinogenic and non-carcinogenic effects of elements in the selected most mobile fractions of PM1—F1 and F2—were analyzed. The exposure concentration (*EC*) for non-carcinogenic and carcinogenic elements in the aforementioned PM1 fractions F1 and F2, respectively, with which an adult and a child contacts via the inhalation route over 24 h was calculated according to the following formula (H. Li et al. 2015):

2 $$ EC=\frac{C\cdot ET\cdot EF\cdot ED}{ATn} $$

where:

*C*—average element concentrations in (F1 + F2) in PM1, μg/m^3^;

*ET*—exposure time, 24 h/day;

*EF*—exposure frequency, days/year;

*ED*—exposure duration, years;

*ATn*—average exposure time, hours.

Time—average period *ATn* for non-carcinogenic elements was calculated on the basis of the formula:

3 $$ ATn= ED\cdot 365\kern0.5em \mathrm{days}/\mathrm{year} $$

In contrast, the average period for carcinogenic elements was determined according to the following formula:

4 $$ ATn=70\kern0.5em \mathrm{years}\cdot 365\kern0.5em \mathrm{days}/\mathrm{year} $$

The risk of exposure to the toxic effect of elements in mobile forms (F1 + F2) contained in PM1 was evaluated by means of so-called hazard quotient (*HQ*) based on the equation proposed in the literature (Biesiada 2001; Xu et al. 2013):

5 $$ HQ=\frac{EC}{RfC\cdot 1000\kern0.5em \upmu \mathrm{g}/\mathrm{mg}} $$

where:

*EC*—exposure concentration, μg/m^3^;

*RfC*—reference concentration, mg/m^3^.

The US EPA website does not quote the value of the reference exposure for Pb; therefore, this element was not taken into account in the assessment based on the so-called *HQ*.

The hazard index (*HI*), which shows the effect of exposure to the influence of a larger number of toxic factors, was determined as a sum of the *HQ* value for particular toxic elements according to the following formula (Trippetta et al. 2015):

6 $$ HI=\sum {HQ}_x $$

where:

*HQ*_*x*_—hazard quotient for element *x*.

When *HQ* and/or *HI* values are below or equal to one, no likelihood of chronic exposure at a given point is assumed, whereas in the case of *HQ* and/or *HI* values above one, the occurrence of hazard caused by toxic substances is probable.

The carcinogenic effect of carcinogenic elements in mobile forms F1 and F2 contained in PM1 was determined on the basis of unit (inhalation) cancer risk. The excess lifetime cancer risk (*ELCR*) via the inhalation route was calculated according to the formula used in literature (H. Li et al. 2015; Wang et al. 2015):

7 $$ ELCR= EC\cdot IUR $$

where:

*EC*—exposure concentration, μg/m^3^;

*IUR*—inhalation unit risk, (μg/m^3^)^−1^.

However, the integrated excess lifetime cancer risk was determined as the sum of *ELCR* values for particular carcinogenic elements (Shaughnessy et al. 2015). According to a common opinion, in the case of carcinogenic elements, a natural safe concentration threshold does not exist and there is always a certain likelihood of cancer. According to the values suggested by the US EPA, the admissible cancer risk occurs within the 1·10^−6^–1·10^−4^ range (H. Li et al. 2015; Sun et al. 2014; Wang et al. 2015). However, the undoubtedly acceptable value is the risk below 1·10^−6^. In the presented investigations, the concentration of Cr in relevant mobile fractions F1 and F2 was determined without determining its particular degree of oxidation Cr(III) or Cr(VI), whereas it is the degree of Cr oxidation that determines its carcinogenicity. According to the guidelines by the US EPA, Cr(VI) is classified as a human carcinogen, whereas Cr(III) is classified as non-carcinogenic to people. Based on the earlier research results (Delgado et al. 2011; Massey et al. 2013; Pui et al. 2014), the authors assumed that the concentration of Cr(VI) was 1/7 of the total concentration of Cr in the relevant fractions F1 and F2.

The results of the assessment of health risk due to inhalation exposure to the toxic effect of elements in mobile fractions F1 and F2 contained in PM1 based on the so-called hazard quotient (*HQ*) for all the measurement points in the surroundings of coking plants (K1–K4) have been presented in Table 3.

**Table 3:** The hazard quotient (*HQ*) of elements contained in the mobile fractions (F1 + F2) of PM1 collected in the surroundings of the four coking plants (K1–K4)

Element	EC (μg/m^3^)				RfC (mg/m^3^)	HQ							
						K1		K2		K3		K4	
	K1	K2	K3	K4		Children	Adults	Children	Adults	Children	Adults	Children	Adults
As	7.18E−04	7.13E−04	9.75E−04	1.27E−03	1.50E−05	4.79E−02	4.79E−02	4.76E−02	4.76E−02	6.50E−02	6.50E−02	8.50E−02	8.50E−02
Cd	4.45E−04	3.75E−04	8.12E−04	1.61E−03	1.00E−05	4.45E−02	4.45E−02	3.75E−02	3.75E−02	8.12E−02	8.12E−02	1.61E−01	1.61E−01
Co	2.48E−05	3.94E−05	2.59E−05	4.83E−05	6.00E−06	4.13E−03	4.13E−03	6.56E−03	6.56E−03	4.31E−03	4.31E−03	8.05E−03	8.05E−03
Cr(VI)	5.50E−05	7.38E−05	7.11E−05	1.05E−04	1.00E−04	5.50E−04	5.50E−04	7.38E−04	7.38E−04	7.11E−04	7.11E−04	1.05E−03	1.05E−03
Hg	6.67E−05	6.84E−05	5.29E−06	5.33E−06	3.00E−04	2.22E−04	2.22E−04	2.28E−04	2.28E−04	1.76E−05	1.76E−05	1.78E−05	1.78E−05
Mn	3.15E−03	6.77E−03	5.84E−03	7.02E−03	5.00E−05	6.30E−02	6.30E−02	1.35E−01	1.35E−01	1.17E−01	1.17E−01	1.40E−01	1.40E−01
Ni	8.75E−04	4.00E−04	5.44E−04	3.29E−04	1.40E−05	6.25E−02	6.25E−02	2.86E−02	2.86E−02	3.88E−02	3.88E−02	2.35E−02	2.35E−02
Sb	8.80E−04	6.12E−04	6.77E−04	1.37E−03	2.00E−04	4.40E−03	4.40E−03	3.06E−03	3.06E−03	3.38E−03	3.38E−03	6.83E−03	6.83E−03
Se	6.47E−04	7.40E−04	7.39E−04	1.27E−03	2.00E−02	3.24E−05	3.24E−05	3.70E−05	3.70E−05	3.70E−05	3.70E−05	6.37E−05	6.37E−05
					HI	2.27E−01	2.27E−01	2.60E−01	2.60E−01	3.10E−01	3.10E−01	4.26E−01	4.26E−01

The values of the hazard quotient calculated separately for each element and hazard index (*HI*) were determined by summing up the *HQ* values for particular elements, in the case of the assumed scenario of inhabitant exposure via the inhalation route, to As, Cd, Co, Cr, Hg, Mn, Ni, Sb and Se absorbed as PM1 at the points in the surroundings of coking plants that do not exceed the safe level of 1 (Table 3). It can be assumed that at the analyzed point in the vicinity of a working coking plant, there is no probability of chronic hazard for children and adults due to the presence of the examined elements in mobile forms (F1 + F2) contained in PM1. In general, the highest values of the total hazard index (4.26·10^−1^) among the examined points were noted at point K4, in the surroundings of a working coking plant. The author’s investigations (Zajusz-Zubek 2016) conducted in Poland in the vicinity of sources of emissions based on coal processing revealed no probability of chronic hazard for children and adults due to the toxic effect of the examined elements in mobile forms (F1 + F2) contained in PM2.5 and PM10. The total hazard index did not exceed the value of 1 at all the examined points (*HI* < 1). In addition, works by Li et al. (2016) revealed no threat (*HI* = 0.5) for children and adults due to exposure to the toxic metals As, Cd, Co, Cr(VI), Mn and Ni in mobile forms (F1 + F2), absorbed via the inhalation route with PM2.5 in the industrial territory of China. In the investigations conducted by Sun et al. (2014), the hazard index (*HI*) exceeded the safe level of 1, reaching a value of 1.86.

For the carcinogenic elements analyzed in this work (As, Cd, Co, Cr, Ni and Pb), the values of potential excess lifetime cancer risk (*ELCR*) and integrated excess lifetime cancer risk (as a sum of *ELCR*) at points (K1–K4) in the surroundings of coking plants have been calculated (Table 4). In the case of carcinogenic elements, the value of natural safe concentration threshold has not been found in the literature and, irrespective of exposure, there is always a certain probability of cancer. The US EPA allows a cancer risk within the range of 1·10^−6^–1·10^−4^. In this work, the authors assumed that the acceptable value of risk is below 1·10^−6^. The presented results show that the potential cancer risk for children, resulting from inhalation exposure to particular carcinogenic elements at points (K1–K4) in the surroundings of coking plants, is below the assumed threshold value (1·10^−6^), whereas the potential cancer risk for adults resulting from inhalation exposure to As and Cr(VI) contained in PM1 was noted at all the points in the surroundings of coking plants. The integrated exposure to the examined carcinogenic elements As, Cd, Co, Cr, Ni and Pb in the case of adults exceeded the admissible limit (1·10^−6^) for PM1 by four times, and in the case of children, by 1.3 times. It has been estimated that for the inhabitants living in the vicinity of coking plants, the integrated excess lifetime cancer risk reached the value of 4.31·10^−6^ for adults and 1.08·10^−6^ for children, i.e., out of one million adults and children living at the analyzed points in the surroundings of coking plants, approximately four adults and one child may potentially develop cancer due to the inhalation exposure to carcinogenic elements contained in PM1. In none of the examined points was the admissible cancer risk (1·10^−6^–1·10^−4^) quoted by the US EPA exceeded. The author’s investigations presented in the work (Zajusz-Zubek 2016) conducted in the surroundings of coking plants (K1–K4) revealed that out of one million adults and children living at the analyzed points in the vicinity of coking plants, approximately seven adults and two children may potentially develop cancer due to the inhalation exposure to the examined carcinogenic elements contained in PM10. The results of research carried out in an urban-industrial territory in Nanjing, China, indicated that carcinogenic elements in mobile forms (F1 + F2) contained in PM2.5 pose a risk of cancer for 11 or even 21 adults out of one million people inhabiting this area (Sun et al. 2014).

**Table 4:** The excess lifetime cancer risk (*ELCR*) of carcinogenic elements contained in the mobile fractions (F1 + F2) via inhalation exposure PM1 in the surroundings of the four coking plants (K1–K4)

Element	EC (μg/m^3^)								IUR ((μg/m^3^)^−1^)	ELCR							
	K1		K2		K3		K4			K1		K2		K3		K4	
	Children	Adults	Children	Adults	Children	Adults	Children	Adults		Children	Adults	Children	Adults	Children	Adults	Children	Adults
As	6.15E−05	2.46E−04	6.12E−05	2.45E−04	8.35E−05	3.34E−04	1.09E−04	4.37E−04	4.30E−03	2.65E−07	1.06E−06	2.63E−07	1.05E−06	3.59E−07	1.44E−06	4.70E−07	1.88E−06
Cd	3.82E−05	1.53E−04	3.22E−05	1.29E−04	6.96E−05	2.78E−04	1.38E−04	5.50E−04	1.80E−03	6.87E−08	2.75E−07	5.79E−08	2.32E−07	1.25E−07	5.01E−07	2.48E−07	9.91E−07
Co	2.12E−06	8.49E−06	3.38E−06	1.35E−05	2.22E−06	8.88E−06	4.14E−06	1.66E−05	9.00E−03	1.91E−08	7.64E−08	3.04E−08	1.22E−07	2.00E−08	7.99E−08	3.73E−08	1.49E−07
Cr(VI)	4.71E−06	1.89E−05	6.33E−06	2.53E−05	6.10E−06	2.44E−05	9.03E−06	3.61E−05	8.40E−02	3.96E−07	1.58E−06	5.31E−07	2.13E−06	5.12E−07	2.05E−06	7.58E−07	3.03E−06
Ni	7.50E−05	3.00E−04	3.43E−05	1.37E−04	4.66E−05	1.86E−04	2.82E−05	1.13E−04	2.40E−04	1.80E−08	7.20E−08	8.24E−09	3.29E−08	1.12E−08	4.47E−08	6.78E−09	2.71E−08
Pb	1.08E−03	4.32E−03	1.01E−03	4.05E−03	1.91E−03	7.65E−03	5.06E−03	2.02E−02	1.20E−05	1.30E−08	5.19E−08	1.21E−08	4.85E−08	2.29E−08	9.18E−08	6.07E−08	2.43E−07
									Sum	7.79E−07	3.12E−06	9.03E−07	3.61E−06	1.05E−06	4.20E−06	1.58E−06	6.32E−06

## Conclusions

The evaluation of mobile and not mobile forms of elements contained in PM1 enables a description of the potential threats resulting from the migration of particular forms of trace elements contained in dust, mainly via the respiratory route. The investigations conducted in the surroundings of coking plants revealed that the average concentration of PM1 was 12.17 (with a range of 3.08–26.37 μg/m^3^). Among the examined elements contained in PM1 in the vicinity of the analyzed coking plants, the highest average percentage (above 50%) in the water-soluble fraction (F1) most dangerous for the environment was noted for As, Cd and Sb. The average percentage contents of the examined carcinogenic and toxic elements in the separated mobile fractions (F1–F3) of PM1 decreased in the following order: As > Pb > Mn > Cd > Sb > Se > Co > Ni > Cr > Hg. The elements that had high percentage contents in the mobile fractions are from anthropogenic sources, which is indicated by their high enrichment factors. A very high risk (RAC above 50%) was found due to the presence of carcinogenic (As and Cd) and potentially carcinogenic elements (Pb and Sb) contained in mobile fractions (F1 + F2) of PM1. The analysis of non-carcinogenic risk based on hazard index *HI* for the assumed scenario of inhabitant exposure revealed that children’s and adults’ absorption of As, Cd, Co, Cr(VI), Hg, Mn, Ni, Sb and Se contained in PM1, which are present in its two most mobile fractions (F1 + F2), can be considered harmless to the health of people living in the vicinity of coking plants in the summer period (*HI* < 1). The results of the conducted evaluation of carcinogenic inhalation exposure to elements contained in fractions (F1 + F2), i.e., As, Cd, Co, Cr(VI), Ni and Pb absorbed with PM1, revealed that approximately four adult persons and one child out of one million people living in the vicinity of coking plants may develop cancer. The results of research presented in the publication can help evaluate a potentially adverse influence on human health on the basis of further toxicological investigations.
